# Electrical/Optical Biosensing and Regulating Technology

**DOI:** 10.3390/bios13060634

**Published:** 2023-06-08

**Authors:** Ning Hu, Hao Wan

**Affiliations:** 1 Department of Chemistry, Zhejiang University, Hangzhou 310058, China; 2 Zhejiang-Israel Joint Laboratory of Self-Assembling Functional Materials, ZJU-Hangzhou Global Scientific and Technological Innovation Center, Zhejiang University, Hangzhou 311215, China; 3 Biosensor National Special Laboratory, Key Laboratory of Biomedical Engineering of Ministry of Education, Department of Biomedical Engineering, Zhejiang University, Hangzhou 310027, China; 4 Binjiang Institute of Zhejiang University, Hangzhou 310053, China

## Introduction

Biosensing has emerged as a powerful tool for exploring biomedical mechanisms. Utilizing highly sensitive electrical and optical sensing technologies, biosensors can detect weak signals and trace biomarkers in a dynamic, real-time, and label-free manner. The current Special Issue of *Biosensors*, “Electrical/Optical Biosensing and Regulating Technology”, presents a collection of papers that describe the latest advances in electrical/optical biosensors and systems. These papers report on the development and application of electrical/optical biosensing and regulating technologies that are expected to significantly enhance biomedical research. By bringing together these cutting-edge technologies and research findings, this Special Issue facilitates the advancement of biosensing and regulatory technology and its impact on the field of biomedical research.

Sun and colleagues have extensively reviewed the application of optical and electrical sensors in flexible wearable wound detection [1]. They emphasize the crucial role of accurately assessing wounds in the treatment process and clarify the physiological significance of typical biochemical indicators and physical parameters in wound healing. Sensors are capable of detecting biochemical markers and physical parameters that reflect wound infection and healing processes, and transmit important physiological information to mobile devices through light or electrical signals. Furthermore, the authors provide a comprehensive overview of the research progress of flexible wearable wound detection sensors based on optical and electrical sensing principles in the past five years, which can help researchers stay up to date with the latest advancements in the field. The challenges and future development prospects discussed in the review can also guide researchers in overcoming current limitations and developing more effective and efficient wearable sensors for wound detection.

Zhan et al. conduct a review of electrochemical sensors and their applications in detecting glycated hemoglobin (HbA1c) [2]. This stable and reliable glycated protein is commonly used to measure glucose levels in diabetes diagnosis. The authors note that the primary performance indicators of electrochemical HbA1c sensors include detection range, detection limit, detection time, sensitivity, and continuous stability. Using these indicators, the authors provide a comprehensive explanation of the principles and performance evaluation of electrochemical HbA1c sensors for various target analytes. They also emphasize that the successful commercial application of HbA1c sensors requires addressing issues such as sensitivity, stability, continuity, and in situ monitoring in complex environments. Overall, this review highlights the importance of electrochemical sensors in biomedical research and their potential to improve the diagnosis and treatment of diabetes.

Microfluidic paper-based analytical devices (μPADs) have greatly promoted the development of in vitro diagnostics due to their low cost, high efficiency, and ease of use. In their review, Zhang et al. provide an overview of the origin, fabrication methods, detection techniques, and innovative applications of μPADs in in vitro diagnostics [3]. They also discuss the portability, sensitivity, and automation of μPADs, and highlight the potential for these devices to be applied in a broader range of medical testing fields in the future. The use of μPADs is an important step forward in improving the accessibility and accuracy of medical testing, and has the potential to greatly benefit patients and healthcare providers alike.

The smartphone-based ratio fluorescence probe (SRFP) platform developed by Wu et al. is promising for the detection and quantification of calcium ions in serum [4]. The use of a 3D printed housing and low-cost optical components makes the platform cost-effective and easy to use. The custom image processing program used to convert the color change of blood calcium into a green-red channel signal ratio linearly related to the concentration of Ca^2+^ is an innovative approach. The detection system has good sensitivity, with a detection limit (LOD) of 1.8 μM in bovine serum samples, and good selectivity, with a recovery rate of 92.8~110.1% and a relative standard deviation (RSD) of 1.72~4.89%. The platform’s advantages of low cost, easy portability, strong operability, high throughput, and good repeatability make it a promising tool for point-of-care testing (POCT) applications.

Yang et al. have developed an innovative platform for high-throughput biochemical analysis that integrates a spectrophotometer with a high-precision ball screw-driven two-dimensional motion slider [5]. The stepper motor-driven slider ensures precise positioning and rapid movement of the microplate, while the compact spectrophotometer and optical path system quickly capture the full-spectrum characteristics of the biochemical reagents. The platform has been proven to exhibit faster measurement speed and higher sensitivity for full-spectrum absorbance of bovine serum albumin (BSA) and glucose solutions, making it a promising solution for high-throughput and full-spectrum biochemical analysis.

Functional near-infrared spectroscopy (fNIRS) is a non-invasive method that can be utilized to detect cerebral hemodynamic responses and reflect the pattern of brain activity under different levels of stress. This technique can be used to assess individual cognitive abilities and psychological/physical health. Bak et al. conducted a study that focused on utilizing the laterality index (LIS) values calculated by fNIRS to differentiate between different types of stress [6]. The authors observed that regardless of whether the stimuli were positive or negative, the eustress group exhibited the largest brain activity, while the distress group showed implicit brain activity. Furthermore, the LIS values of the stress group, control group, and distressed group were sequentially larger. These findings suggest that the stress group can be further divided into eustress and distress groups, which lays the foundation for using fNIRS to subdivide the stress group into different types. Therefore, fNIRS is a promising tool for assessing stress and its different types.

Liang et al. have proposed a novel method for quantitatively analyzing global DNA methylation using methylation-specific antibodies (5mC) modified magnetic beads (MB) for immune recognition and affinity enrichment [7]. The method is based on the catalytic reaction product of the interaction between the DNA captured on the MBs surface and the DNA antibody, which is then transferred to a lead-doped screen-printed electrode for electrochemical detection of the overall level of DNA methylation under the catalysis of glucose oxidase. This approach has been successfully applied to three different hepatocellular carcinoma (HCC) cell lines and has demonstrated the ability to detect methylation levels as low as 5% within 70 min. This method holds great promise for both academic research and clinical applications.

Terahertz radiation is a relatively new and unique radiation source that has been widely applied in various fields. In a recent study by Qi et al., the effects of 0.14 THz terahertz radiation on mouse behavior were investigated [8]. The study utilized a range of behavioral tests, including open field experiment, elevated plus maze experiment, light-dark box experiment, three-chamber social experiment, and forced swimming experiment. The results showed that terahertz waves can enhance the anti-anxiety, anti-depressive, and social interaction abilities of mice. These findings have significant practical implications for studying the effects of terahertz radiation on mouse behavior.

Electrical/optical biosensing and regulating technology can provide accurate and real-time measurements of various biological and environmental parameters, which can help researchers and scientists better understand complex biological systems and environmental processes. This Special Issue showcases the progress and potential applications of different sensor technologies in various fields, opening up new avenues for the future development of biosensing.
